# Correlations between Diffusion Tensor Imaging (DTI) and Magnetic Resonance Spectroscopy (^1^H MRS) in schizophrenic patients and normal controls

**DOI:** 10.1186/1471-244X-7-25

**Published:** 2007-06-19

**Authors:** Cheuk Y Tang, Joseph Friedman, Dikoma Shungu, Linda Chang, Thomas Ernst, Daniel Stewart, Arash Hajianpour, David Carpenter, Johnny Ng, Xiangling Mao, Patrick R Hof, Monte S Buchsbaum, Kenneth Davis, Jack M Gorman

**Affiliations:** 1 Department of Radiology, Mount Sinai School of Medicine NY, NY 10029, USA; 2 Department of Psychiatry, Mount Sinai School of Medicine NY, NY 10029, USA; 3 Department of Neuroscience, Mount Sinai School of Medicine NY, NY 10029, USA; 4 Department of Pathology, Mount Sinai School of Medicine NY, NY 10029, USA; 5 Department of Radiology, Weill Medical College of Cornell University, NY, NY 10021, USA; 6 Department of Medicine, University of Hawaii, HI 96817, USA

## Abstract

**Background:**

Evidence suggests that white matter integrity may play an underlying pathophysiological role in schizophrenia. N-acetylaspartate (NAA), as measured by Magnetic Resonance Spectroscopy (MRS), is a neuronal marker and is decreased in white matter lesions and regions of axonal loss. It has also been found to be reduced in the prefrontal and temporal regions in patients with schizophrenia. Diffusion Tensor Imaging (DTI) allows one to measure the orientations of axonal tracts as well as the coherence of axonal bundles. DTI is thus sensitive to demyelination and other structural abnormalities. DTI has also shown abnormalities in these regions.

**Methods:**

MRS and DTI were obtained on 42 healthy subjects and 40 subjects with schizophrenia. The data was analyzed using regions of interests in the Dorso-Lateral Prefrontal white matter, Medial Temporal white matter and Occipital white matter using both imaging modalities.

**Results:**

NAA was significantly reduced in the patient population in the Medial Temporal regions. DTI anisotropy indices were also reduced in the same Medial Temporal regions. NAA and DTI-anisotropy indices were also correlated in the left medial temporal region.

**Conclusion:**

Our results implicate defects in the medial temporal white matter in patients with schizophrenia. Moreover, MRS and DTI are complementary modalities for the study of white matter disruptions in patients with schizophrenia.

## Background

Alterations in connectivity between brain regions including the frontal lobe, basal forebrain and limbic system, have been proposed as network deficits in schizophrenia [1-6]. Connections between the prefrontal cortex (PFC) and other cortical and subcortical regions implicated in the pathophysiology of schizophrenia suggest that a white matter abnormality in this region could have widespread consequences for neural connectivity in brain regions that are critical to the core symptoms of schizophrenia. The finding of increased neuronal density [7-13] is consistent with the notion that the integrity of white matter tracts is compromised. In addition, MRI evidence for decreased global [14-16], prefrontal [15,17-21] and temporal [15,21] white matter in schizophrenia is quite convincing. A meta-analysis of studies of white matter volumes in schizophrenia reveals a reduction of approximately 2%, with the greatest differences of approximately 5% in medial temporal lobe structures [14]. Indeed, regional rather than global reductions are far more relevant to the question of the origins of disconnectivity and hence of particular interest in the presentation of symptoms. For example, volume reductions in the white matter of PFC, repeatedly found in schizophrenia [17-20], are associated with the presentation of negative symptoms [18,20].

Both MRS [22,39,41,42,44,46-27] and DTI [28-34] have been used extensively to study white matter abnormalities in patients with schizophrenia. The results of these two techniques may reflect different mechanisms of abnormal pathologies. MRS measurements are determined by biochemical profiles of the underlying pathologies, whereas DTI is more sensitive to structural differences.

Magnetic resonance spectroscopy allows one to quantify the concentrations of various metabolites in the brain. N-acetylaspartate (NAA) is the most prominent metabolite detected in the normal human brain. NAA serves as a neuronal marker and is only present in mature neurons. Some studies have also shown the presence of NAA in oligodendrocyte-2A progenitor cells [35]. NAA has been shown to be decreased in well-known demyelinating diseases such as multiple sclerosis (MS) and progressive multifocal leukoencephalopathy (PML) and it is thought to be associated with axonal injury [36]. Decreased NAA in patients with schizophrenia has been found in the cerebellum [37], thalamus [23,38-44], dorso-lateral prefrontal cortex (DLPFC) [23,42-44], anterior cingulate [45], and medial temporal lobe [24-27,46,47]. Reduction in NAA may signify structural abnormality or reduced viability of the underlying neurons [35,48]. Given these results we have acquired MRS data on the DLPFC white matter, medial temporal (MT) white matter and on the occipital (OC) white matter as a reference.

Diffusion tensor imaging allows one to quantify the integrity of densely packed fiber bundles such as axonal tracts and to measure the orientation of such bundles [49-53]. DTI probes the microstructure of white matter by measuring the anisotropy of self-diffusion of water molecules in the restricted compartments of axonal tracts. Two quantitative measures can be obtained from DTI: anisotropy indices and fiber tract orientations. Anisotropy indices such as relative anisotropy (RA) and fractional anisotropy (FA) [49,53] measure the amount of coherence of water diffusion which putatively reflects the amount of myelination in axonal bundles or the coherence of fiber tracts. The same dataset also provides information on the three-dimensional orientation of the anisotropy and can be used to study fiber tract connectivity [54-56]. Previous DTI studies have shown reduced anisotropy in patients with schizophrenia in frontal white matter [28,29,57,58], the cingulum bundle [31,59], the temporal gyrus [32,60], and the corpus callosum [32,34,61].

Given that MRS and DTI can provide complementary imaging data on white matter changes in brain we sought to investigate the white matter brain changes associated with schizophrenia by simultaneously acquiring DTI and MRS data in a cohort of schizophrenic patients and a group of matched healthy control subjects. MRS was acquired targeting three regions of interests: frontal white matter, occipital white matter and medial temporal white matter. Since the DTI scans are much less time-consuming than MRS scans, we acquired whole brain DTI and extracted matching voxels for correlation analysis with the MRS voxels.

## Methods

### Subjects

Schizophrenic subjects were recruited from inpatient, outpatient, day treatment and vocational rehabilitation services at Mount Sinai Hospital (New York, N.Y.), Pilgrim Psychiatric Center (W. Brentwood, N.Y.), Bronx VA Medical Center (Bronx, N.Y.), Hudson Valley Veterans Affairs Medical Center (Montrose, N.Y.), and Queens Hospital Center (Jamaica, N.Y.) following approvals by each institutional review board. Informed consent was obtained on each subject following an assessment of capacity to provide informed consent by a psychiatrist independent of the study. The inclusion criteria were 1) a DSMIV diagnosis of schizophrenia or schizoaffective disorder based on the Comprehensive Assessment of Symptoms And History (CASH) [62], and 2) aged 18–80. Healthy comparison subjects who were without any DSMIV axis I disorder (by CASH interview) were recruited from the New York area and were matched for age and gender to the schizophrenic subjects, they also provided informed consent. Subjects were excluded if they had 1) a positive urine drugs of abuse screen, 2) a medical diagnosis which may produce white matter changes (i.e. HIV, MS), 3) a history of brain disorder which may produce cognitive impairment or behavioral symptoms (i.e. head injury, cerebrovascular disease), or 4) had an unstable medical condition (i.e. poorly controlled diabetes or hypertension, symptomatic coronary artery disease). Each subject was carefully screened and evaluated with a neuropsychological evaluation, structured assessment of symptoms, screening medical history, physical examination and laboratory studies (including complete blood count, routine chemistry, liver enzymes, and thyroid function tests) and urine toxicology screen, to ensure they fulfilled the inclusion criteria.

42 schizophrenic subjects and 40 healthy comparison subjects were recruited, assessed and scanned with both DTI and MRS modalities. Mean age of the schizophrenic group did not differ significantly from the healthy comparison group (schizophrenic mean = 38.69 years [sd = 11.42]), healthy comparison mean = 43.3 years [sd = 20.18], t = 1.28, p = .20), nor did the gender distribution (healthy comparison males = 57.5%, schizophrenic males = 69%, X^2 ^= 1.18, p = .28). However, schizophrenic subjects attained significantly lower levels of education than the healthy comparison subjects (schizophrenic mean = 12.21 years [sd = 2.07]), healthy comparison mean = 15.3 years [sd = 2.24], t = 6.48, p < .001). The average age of illness onset for the schizophrenic group was 23.53 years (sd = 7.09), 5% of the schizophrenic sample was not receiving treatment with any antipsychotic at the time of scanning while 19% were receiving first generation antipsychotics, 17% clozapine, and 59% were receiving other second generation antipsychotics (risperidone, olanzapine, ziprasidone, quetiapine, aipiprazole).

### Data Acquisition

All imaging studies were performed on an Allegra 3T head-dedicated system (Siemens, Ehrlangen, Germany) with a gradient strength of 40 mT/m and slew rate of 400~900 mT/m/s, allowing EPI acquisitions with minimal susceptibility distortions. Patient head motion was minimized by inflatable pillows inserted between the patients' head and the head coil. Subjects were recruited to receive two scans, one MRS and one structural. Most subjects received both scans on the same day, but there were some that preferred to return on a second day for the other scan. The duration between the two scans was at most 2 months apart. During this time there was no change in treatment that the patients received.

#### MRS

**Figure 1:**
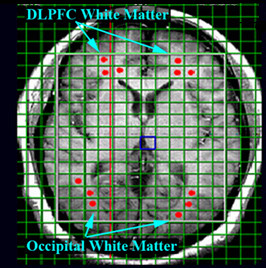
T1-weighted axial slice for DLPF wm and OCC wm regions with CSI acquisition matrix and ROIs for metabolites quantification superimposed.

**Figure 2:**
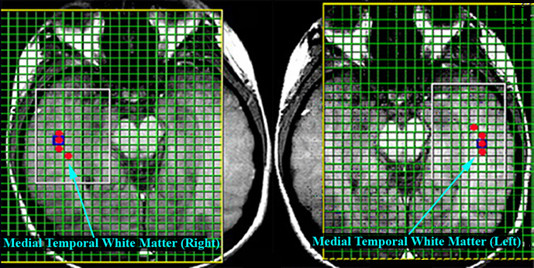
T1-weighted axial slices for left and right MT wm regions with CSI acquisition matrix superimposed as well as ROIs for metabolites quantification.

Localizer MR images for prescribing the MRS volumes consisted of a T1 sagittal with the following parameters: TR = 500 ms, TE = 10 ms, FOV = 18 cm × 14 cm, matrix size = 512 × 384, 4.3 mm thick with 1.1 mm spacing. 25 slices were obtained to cover the whole brain. From these sagittal images, two T1-weighted transverse slices (TR = 500 ms, TE = 10 ms, Thickness = 10 mm, FOV = 16.5 cm × 22 cm with matrix 512 × 384) were identified for MRS acquisition: the first slice covered the DLPFC white matter and the OC white matter. A nearly axial plane was chosen for the plane going through the striatum. It is chosen to be parallel to the AC-PC line as identified on the sagittal planes (Fig 1). The second slice was prescribed to be co-axial with the Sylvian fissure such that it contained the medial temporal lobe white matter bilaterally (Fig 2). 1H spectroscopic imaging (SI) data of the left and right medial temporal lobes were obtained in two sequential scans using the phase-encoded version of the standard PRESS volume localization sequence, with TR = 2000 ms, TE = 30 ms, 24 × 24 phase-encoding steps over a field-of-view of 16 cm (zerofilled to 32 × 32 phase-encoding steps before 3D Fourier transformation), a slice thickness of 10 cm slice, 1 average per phase-encoding step and circular k-space sampling, to obtain voxels having a nominal size of 0.25 cm3 (1.0 × 0.5 × 0.5 cm^3^). Outer volume saturation bands were prescribed to coincide with all 8 sides of the PRESS box. Water suppression and magnet shimming were automatically performed and adjusted by the host computer. The 1H SI data for the DLPFC were acquired with the same protocol except that the FOV changed to 30 cm. The resulting interpolated pixel size was (1.0 × 0.9 × 09 cm^3^). One slice was acquired for the DLPFC and OC white matter tracts. Total imaging time for all three slices averaged about 75 minutes.

**Figure 3:**
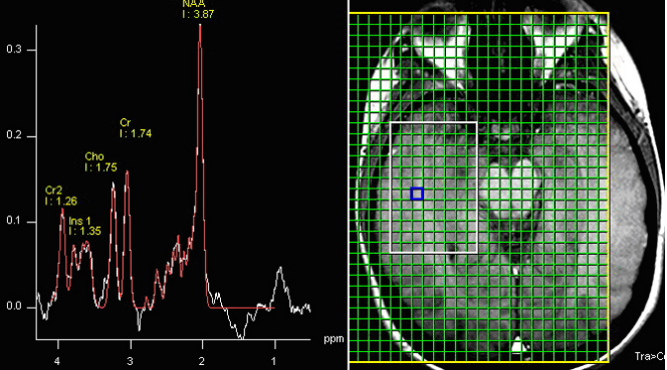
Example of MRS spectrum of a MT wm voxel (blue square) extracted from a 2D-CSI acquisition. Visible metabolite peaks shown are NAA, Cr, Cho, Ins1 and Ins2.

The raw SI data were processed and fitted in the frequency-domain to obtain metabolite peak areas using manufacturer-supplied MRS data processing software. Individual CSI images were reconstructed and overlaid onto the T1 anatomical images. Automatic phase correction was applied, voxels of interests were identified, and the metabolite levels were derived from the spectral fits. For the DLPFC white matter and OC white matter ROIs, three voxels were identified visually (Fig 1) for each hemisphere. For the MT white matter four voxels were identified per hemisphere because of the higher CSI resolution (Fig 2). MRS metabolites (NAA, Cho, Cr, Ins1 & Ins2) were obtained from these regions of interests (ROIs) (Fig 3). The number of voxels per ROI was variable ranging between 1 and 3. Voxels with poor spectral data quality, defined asunresolved Cr and Cho resonances, were excluded from analysis. The metabolites from the selected voxels in each of the ROIs were averaged and transferred to Statistica v6 (StatSoft Inc., Tulsa, Ok) for statistical analysis.

#### DTI

DTI data was acquired in a separate scanning session that included other structural scans for morphological analysis. The protocol for the structural scans consisted of a three-plane sagittal localizer from which all other structural scans were prescribed. The following structural scans were acquired: Axial 3D-MPRage (TR = 2500 ms, TE = 4.4 ms, FOV = 21 cm, matrix size = 256 × 256, 208 slices with thickness = 0.82 mm); Turbo spin echo T2-weighted Axial (TR = 5380 ms, TE = 99 ms, FOV = 18.3 cm × 21 cm, matrix = 512 × 448, Turbo factor = 11, 28 slices, thickness = 3 mm skip 1 mm); DTI using a pulsed-gradient spin-echo sequence with EPI-acquisition (TR = 4100 ms, TE = 80 ms, FOV = 21 cm, matrix = 128 × 128, 28 slices, thickness = 3 mm skip 1 mm, b-factor = 1250 s/mm^2^, 12 gradient directions, 5 averages); and magnetization transfer imaging (MTI) using a turbo spin echo sequence (TR = 700 ms, TE = 12 ms, FOV = 21 cm, Matrix = 256 × 256, 28 slices, thickness = 3 mm skip 1 mm). Total imaging time for the structural scans averaged about 50 minutes.

**Figure 4:**
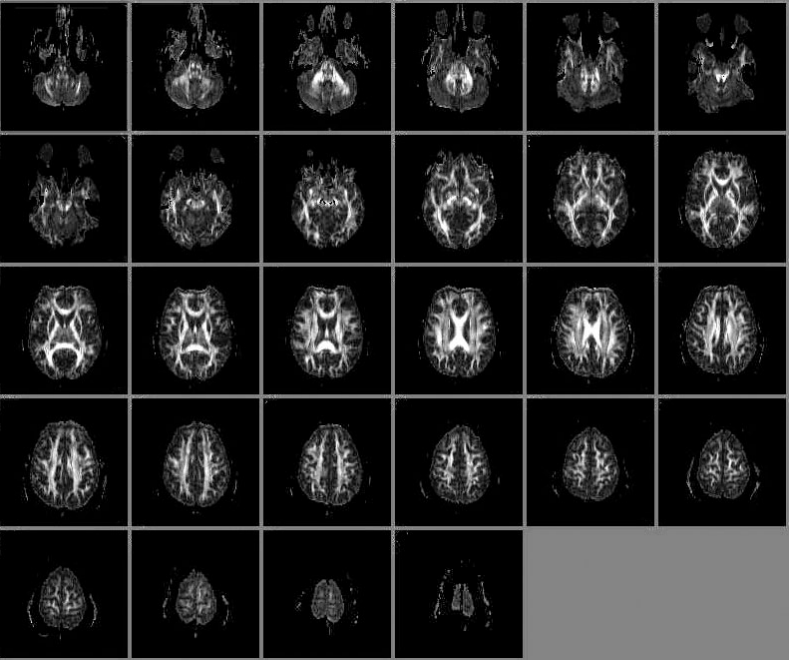
Diffusion Tensor Imaging: Whole-Brain Fractional Anisotropy Maps of normal control.

**Figure 5:**
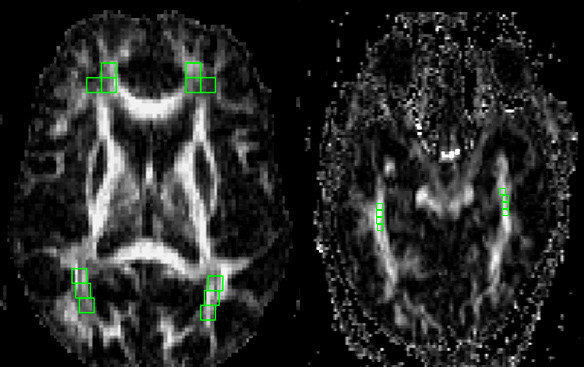
ROI locations (left) DLPF wm, OCC wm and (right) MT wm for Fractional Anisotropy Data.

Raw DTI data were transferred to an off-line workstation for post-processing. In-house software written in *Matlab *v6.5 (The Mathworks Inc. Natick, MA) was used to compute the anisotropy and vector maps. The Fractional Anisotropy images were then converted to analyze format (Fig 4). *MEDx *v3.4.3 software (Medical Numerics Inc, Sterling, VA) was used to inspect and define ROIs on the FA images. Two adjacent axial slices (4 mm) were selected from the FA dataset for each matching CSI image (10 mm). The planes were selected to contain the same CSI voxel locations (Fig 5). The voxels for the FA images measured 1.6 × 1.6 × 3 mm^3^. ROI dimensions for the FA images were set to 5 × 5 and 3 × 3 for the DLPFC/OC and MT respectively. These settings gave us the closest match to the MRSI data in terms of actual volumes sampled; ROI dimensions for DLPFC and OC were 8.2 mm (FA) vs. 9 mm (MRSI), ROI dimensions for the MT were 4.9 mm (FA) vs. 5 mm (MRSI). For all structures 2 FA slices were sampled corresponding to a thickness of 8 mm (FA) vs. 10 mm (MRSI). The FA voxel statistics for each ROI were extracted and transferred to *Statistica *and merged with MRS data for t-tests and correlation analysis.

In order to ensure reliability of our region of interest procedures inter-rater reliability measures were calculated for the two raters for each white matter region in 20 subjects. The intraclass correlation coefficients on the mean values of the ROIs as obtained by the two raters were as follows; optic radiations (right = .83, left = .83), MT (right = .88, left = .91), DLPF WM (right = .94, left = .84).

## Results

Statistical tests between the average ROI values were performed on the fractional anisotropy as well as the MRSI data for the three regions of interests in both hemispheres. Table 1 shows the mean fractional anisotropy and metabolite ratio values for the schizophrenic and healthy comparison groups in each medial temporal regions of interest. Independent T-tests on the metabolite ratios demonstrated significantly reduced NAA in the left medial temporal white matter regions in patients with schizophrenic subjects (mean = 1.17) compared to healthy control subjects (mean = 1.31) (t = 2.35, df = 75, p < .021). Similar significances were found in the right medial temporal white matter regions with schizophrenic subjects (mean = 1.07) and healthy control subjects (mean = 1.21) (t = 2.29, df = 75, p < .025). We also computed the significances for NAA/Cr ratios in the medial temporal white matter region. Significant differences were found in the left with schizophrenic subjects (mean = 1.96) and healthy controls (mean = 2.37) (t = 2.58, df = 75, p < 0.012) as well as on the right with schizophrenic subjects (mean = 1.94) and healthy controls (mean = 2.25) (t = 2.17, df = 75, p < 0.033). Other areas and metabolite concentrations were not significantly different between groups.

**Table 1:** Mean values of metabolite ratios and fractional anisotropy (FA) and for the Medial Temporal, DLPFC and Occipital white matter ROIs. Significant differences between schizophrenic and healthy control subject groups by independent t tests are highlighted in bold.

	**NAA**			**NAA/Cr**			**FA**		
**ROI**	**Schizophrenic Mean (SD)**	**Control Mean (SD)**	**p**	**Schizophrenic Mean (SD)**	**Control Mean (SD)**	**p**	**Schizophrenic Mean (SD)**	**Control Mean (SD)**	**p**

**R Medial Temporal**	**1.076(0.233)**	**1.210(0.276)**	**<0.025**	**1.937(0.556)**	**2.254(0.716)**	**<0.033**	**0.307 (0.052)**	**0.342(0.073)**	**<0.003**
**L Medial Temporal**	**1.171(0.273)**	**1.308(0.238)**	**<0.021**	**1.956(0.732)**	**2.368(0.732)**	**<0.012**	**0.370 (0.082)**	**0.339(0.064)**	**<0.023**
**R Frontal**	4.260(1.420)	3.970(1.230)	p = 0.38	2.290(0.770)	1.990(0.610)	p = 0.08	0.230 (0.043)	0.244 (0.055)	p = 0.21
**L Frontal**	4.550(1.440)	4.450(1.330)	p = 0.77	2.320(0.730)	2.280(1.080)	p = 0.88	0.226(0.044)	0.237(0.052)	p = 0.30
**R Occipital**	4.050(2.240)	4.070(1.320)	p = 0.96	3.870(2.540)	2.360(0.780)	p = 0.30	0.315(0.045)	0.325(0.051)	p = 0.36
**L Occipital**	4.360(2.640)	4.270(1.520)	p = 0.87	2.840(1.830)	2.520(0.730)	p = 0.34	0.356(0.069)	0.364(0.059)	p = 0.60

T-tests on the matching voxels of the FA data showed significantly reduced FA values in the same right medial temporal white matter voxels as the MRS in patients with schizophrenia compared to normal control subjects (0.307 vs. 0.342) (t = 3.02, df = 79, p < .003). FA was also significantly reduced in the left medial temporal white matter region of schizophrenic subjects compared to healthy control subjects (0.339 vs. 0.370) (t = 2.31, df = 79, p < .023). Other areas surveyed were not significant. Correlation analysis on combined patient and control subjects showed a significant correlation between FA and NAA in the left medial temporal (r = 0.210, p = .050) as well as FA and NAA/Cr (r = 0.230, p = 0.040). No significant correlation was found in the right medial temporal side (r = 0.210, p = 0.090) and (r = 0.140, p = 0.220). There have been several reports suggesting that additional neuro-degenerative factors such as age might have effects on FA [63,64]. NAA in our patient sample as well as some other studies [65] did not show any age effect. We have also tested partial correlations with the FA values corrected for age: a slight increase in significances were found in the same regions, correlations between FA and NAA, NAA/Cr in the left medial temporal white matter were (r = 0.332., p = .010) and (r = 0.308, p = 0.017) respectively. The correlations in all other ROIs surveyed were not significant: right medial temporal side (r = 0.20, p = 0.07) and (r = 0.16, p = 0.22); left DLPFC (r = 0.03, p = 0.80) and (r = -0.07, p = 0.56), right DLPFC (r = 0.07, p = 0.56) and (r = 0.23, p = 0.06), left occipital (r = 0.13, p = 0.10) and (r = 0.15, p = 0.21), right occipital (r = 0.10, p = 0.39) and (r = 0.18, p = 0.13) respectively.

## Discussion

The regions sampled in the current study shows that NAA/Cr and fractional anisotropy are reduced in the medial temporal white matter in patients with schizophrenia. Reduced anisotropy was detected in the areas surveyed despite normal appearing white matter on conventional T1 and T2 weighted images which suggest microscopic damage to these fiber tracts.

NAA is thought to be present almost exclusively in neurons and their dendritic and axonal extensions but not in glia [66-68]. The NAA signal provides a marker of the number of viable neurons [69]. NAA is an intracellular amino acid derivative produced in the mitochondria [70], but it is also found in large quantities in O-2A progenitor cells [35]. O2-A progenitor cells are putatively involved with glia repair processes[71]. MS studies have shown that it is the failure of these repair processes that is the main cause of the disease [72]. A reduction in NAA may reflect both a volume loss as well as a defect in the myelin maintenance infrastructure. FA indices from DTI measurements reflect the amount of coherently restricted diffusion (imposed by the presence of myelin) of free water. These coherently restricted diffusion pathways are most prominent in axonal bundles. DTI voxels are several orders of magnitude larger than cellular dimensions, so that the computed anisotropy indices reflect the cumulative effect of the underlying microstructure. While there is still controversy regarding the source of the anisotropy such as the contribution of the intracellular versus extracellular water to the diffusion signal [73-76], one can argue from a physical point of view that a reduced anisotropy can be the result of one of the following phenomenon: loss of myelin leading to reduced restricted diffusion, intact fibers but not coherently oriented and loss of fibers. Measurements using multiple b-values have shown a dependence of the diffusion signal on fast and slow diffusion as well as a deviation from mono-exponential signal decay with higher b-values. With b-values below 2000 s/mm^2 ^the signal decays mono-exponentially and is most sensitive to fast diffusion [74]. Our DTI data was acquired with a b-value of 1250 s/mm^2 ^and is thus sensitized to fast diffusing water protons (probably in the extra-cellular space).

In a systematic review of proton MRS investigations measuring NAA differences between healthy controls and patients with schizophrenia [77] levels of NAA do appear to be substantially reduced in frontal WM and temporal WM of schizophrenic patients. Although studies of temporal WM NAA changes are less well corroborated than frontal WM changes in schizophrenia the mean reduction of FA in temporal WM across studies was estimated at 12.7% compared to 5.2% for frontal WM [77]. Moreover, antipsychotic treatment of the patients in our study may have confounded the measurements of NAA in the prefrontal white matter. In a longitudinal evaluation chronic schizophrenic patients first scanned off medication then after treatment was started NAA levels increased significantly and selectively in the dorsolateral prefrontal cortex within 4 weeks but not in temporal lobe or other areas assessed [42]. The fact that we only found significant differences in the medial temporal white matter may support these findings.

Our group has previously published a report on the DTI findings of the same subject population [78]. SPM analysis showed that there were significant differences in the medial temporal white matter as well as frontal white matter regions. The areas of significance were in the same locations as our ROI placements. The reason that we did not find significant FA differences in the frontal white matter is partly due to the size of the ROIs. The ROI dimensions and locations chosen for the current analysis was based on the need to match the voxels obtained from the MRS data. These results suggest that axonal disruptions in the frontal white matter areas might be limited to specific tracts within the frontal white matter. The subjects used for the current analysis is a subset of the subjects used in our previous report (N = 55, S = 63) versus (N42, S = 40) because some scans had to be eliminated due to poorly resolved spectra.

A similar study was performed by Steel et. al. [79], but no significant differences were found in NAA or FA. This might have been due to several reasons. Single voxel MRS is prone to partial volume contamination. Our technique used multivoxel spectroscopic imaging with much smaller voxel size; we only sampled the voxels which are well contained within the white matter regions. The second reason for the difference in findings may be due to our much larger sample of subjects (n = 40/42 vs. n = 10/10) and potential mean age differences (Steel's group = 35/34, this study = 43/39). The Steel's group did not survey the medial temporal lobe which was the location where we found significances for both NAA and FA.

A more recent study has been performed by Irwan et. al. [80] using DTI and CSI acquired in a supraventricular slice in normal control subjects. Although they have not specifically looked at isolated white matter voxels, they have shown a significant positive correlation between NAA and FA as well as a significant negative correlation between NAA with ADC values which is consistent with our interpretation of the FA index.

We also found significant correlations between FA and NAA in the left medial temporal white matter. Despite the co-occurrence of the reductions in NAA and fractional anisotropy these values were not significantly correlated on the right side (p = 0.07). These relatively weak significances were only observed when correlations were computed with both control subjects and patients combined. Therefore, these results between these two measures in the same region may be suggestive of different structural and/or metabolic changes co-occurring in the same region, but independent of each other, in contrast to what is observed in multiple sclerosis [81]. For example, patients with schizophrenia may be born with proper axonal formation but defective myelin maintenance infrastructure as reflected by reduced NAA/Cr. This interpretation is reasonable given that NAA is also found in large quantities in O-2A progenitor cells [35] putatively involved with glia repair processes [71]. In this scenario our results could be interpreted such that the reduction of anisotropy in this area might occur gradually whereas the low NAA/Cr levels could be constant throughout the lifespan of the patients. Low NAA may be a hallmark throughout the lifespan of the disease but its effect and other factors on FA may be more gradual.

One limitation of this study lies in the coregistration of the MRS ROIs with the DTI ROIs. Due to the lower sensitivity of MRS, the acquisition of MRS data is inherently slower and the resulting data is of lower resolution. We were only able to acquire two slices of MRS data. This made it impossible to accurately coregister the MRS slices to the DTI slices using conventional computer algorithms. The problem is compounded by the inherent distortions that exists in EPI based DTI acquisition schemes. In this study we have relied on our anatomy experts to visually identify the regions of interests. To minimize the error we have acquired the MRS slice for the DLPFC and the occipital regions in the same plane as the DTI which was along the AC-PC plane. This made it easier to identify the same regions on both modalities. Other limitations are the differences in slice thickness between the two modalities. The MRS was 10 mm thick whereas the DTI were 3 mm skip 1 mm. To minimize this difference we selected two DTI slices for every MRS slice.

## Conclusion

Our study has provided further evidence of white matter abnormality in the medial temporal region in chronic schizophrenic patients. This was evidenced through MR Spectroscopy as well as Diffusion Tensor Imaging. Applying these two imaging modalities to an at risk population at a younger age before the disease is diagnosed and correlating the results with outcome would elucidate the sequence of events such as defective NAA preceding reduced FA. Differences in the MRS and DTI results may help one distinguish the nature of the white matter defects.

## Competing interests

The author(s) declare that they have no competing interests.

## Authors' contributions

CYT was responsible for the DTI software development, MRI protocols, analysis and interpretation of the results. JF and DS was responsible for the recruitment and screening of the subjects. LC, TE and DS were responsible for the MRS protocols. AH, DC, JN and XM were responsible for ROI tracings. PRH was responsible for the anatomical localization of the ROIs.

The study was conceived by KD, MSB and JMG. All authors have read and approved the final manuscript.

## Pre-publication history

The pre-publication history for this paper can be accessed here:


